# Editorial: From simulation to the operating theatre: new insights in translational surgery

**DOI:** 10.3389/fmedt.2023.1282248

**Published:** 2023-09-21

**Authors:** Naci Balak, Eleni Tsianaka, Cesare Zoia, Amitendu Sekhar, Mario Ganau

**Affiliations:** ^1^ Department of Neurosurgery, Istanbul Medeniyet University, Göztepe Hospital, Istanbul, Türkiye; ^2^ Neurosurgery Department, Kuwait Hospital, Sabah Al Salem, Kuwait; ^3^ Neurosurgery Unit, Ospedale Moriggia Pelascini, Gravedona, Italy; ^4^ Department of Neurosurgery, Bahrain Defence Force Royal Medical Services Military Hospital, West Riffa, Bahrain; ^5^ Nuffield Department of Neurosciences, University of Oxford, Oxford, United Kingdom

**Keywords:** microsurgical laboratory, operating theater, surgical simulation, surgical simulation models, virtual reality, surgery, surgical laboratory, surgical innovation

**Editorial on the Research Topic** From simulation to the operating theatre: new insights in translational surgery

Technological tools such as highly advanced surgical microscopes, complex microsurgery laboratories, virtual reality, 3-D printing, neuronavigation, surgical robotics, and artificial intelligence (AI) have already become a part of daily life in many surgical specialties. To improve the quality of treatment provided in operating rooms, it is essential that such technologies be adapted to every surgical branch in a timely manner and that surgeons become familiar with them in the surgical laboratory (1). In this way, new scientific and technological developments will be translated for implementation not only in real-life interventions but also in surgical training programs (2). The objective of this Research Topic, therefore, is to bring together surgeons and basic scientists working at the forefront of innovative technologies intended to transform surgical practice. This editorial will summarize the findings of the articles included in this Research Topic.

Three articles in this Research Topic are dedicated to surgical simulation. Culmone et al. report on the development of a new, purely mechanical, programmable physical track called MemoBox to control endoscope-like surgical instruments. They also show the possibility of creating an endoscope with a highly controllable, multisteerable, and precise shaft. The design of advanced snake-like surgical instruments capable of memorizing three-dimensional pathways represents a remarkable step forward in many surgical scenarios, such as colonoscopy, interventional bronchoscopy, or skull base and spinal surgery. The latter subspecialties are particularly challenging because the structures around the surgical channel might be extremely fragile and susceptible to iatrogenic injuries (3). Hence, the learning experience of MemoBox may prove a game changer and expand the horizons of surgical practice. Three-dimensional printing is another groundbreaking technology that promises to enable simulation applications with patient-specific models. In this regard, Takoutsing et al. evaluate the effects of using a new virtual-physical modeling/simulation application tool called UpSurgeOn on the neurosurgery and neuroanatomy training of medical students in Africa. The authors reveal that the use of non-cadaver 3D-printed models could contribute to improving the psychomotor performance of surgeons in microsurgery. The authors also note that most medical students became more familiar with neurosurgery after using this tool; the students gained neurosurgical skills and developed the orientation skills needed during neurosurgical applications. In addition, according to the study, students expressed their satisfaction with the integration of this method into their medical curriculum. Beyond medical education and basic surgical training, the art of surgery requires lifelong learning, creativity, and subspecialization (4–7), as confirmed by Kil et al., who present a simulator for the assessment of suturing skills. They were able to collect data on hand force, motion, and touch applied while suturing to gauge surgeons' skills and dexterity. Combining these data with video information, the vision-activated force measurements provided deeper insights into suturing performance, especially for forces perpendicular and tangential to the suturing direction. Overall, these three studies align well with the surgical community's efforts to accelerate the training curriculum through exercises dedicated to skill acquisition (8). The results of recent surveys sponsored by national and international societies have demonstrated the value of doing so (9) and the COVID-19 pandemic provided opportunities for implementing such methodologies to maintain skills and ensure preparedness for real-life surgery (10).

This Research Topic also confirms the keen attention being paid by the surgical community to digital medicine and the use of data analysis for surgical planning (11, 12). The current landscape of AI in surgical simulations is outlined comprehensively by Park et al. The authors provide numerous examples that demonstrate the effectiveness of AI-powered surgical simulators in clinical practice. Another article from this Research Topic by Zhu et al. presents a successful example of the application of digital technology and finite element analysis in spinal deformities associated with ankylosing spondylitis. Both studies have the merit of using AI-powered prediction models for surgical planning, and they both pivot on examples recently approved or under evaluation by regulatory agencies.

Finally, beyond the educational roles and use as planning tools discussed above, we need to recognize that emerging technologies are already reducing the cost of medical care. Encarnacion Ramirez et al. studied the effectiveness of using a self-made, low-cost exoscope as an alternative to a conventional operating microscope in anterior cervical discectomy and fusion. The authors conclude that the exoscope is a safe and effective alternative with great ergonomics, comfort, simplicity, and low-cost advantages; hence, it could expand the options for low-cost microsurgery in less resourced countries.

In summary, it is important to highlight that this Research Topic, including four original research studies, one clinical trial, and one perspective article, has important implications for patient safety. Surgical safety is a global health issue; unsafe surgery leads to increased patient morbidity/mortality, legal claims against surgeons, and a distrust of physicians (13, 14). The evolution of simulators in surgical specialties promises to have a positive impact on the training of residents and patient safety, and the use of new technologies discussed in this Research Topic to provide better care in low- and middle-income countries represents a source of hope for the future of our global surgery community. While a roadmap for future endeavors in surgical practice should also touch upon other groundbreaking discoveries in the field of basic sciences, such as nanomedicine and biomedical engineering, which are translatable to the field of intraoperative imaging (15) and postoperative therapies (16), the articles included in this Research Topic allow us to obtain a rich overview on equally relevant aspects of digital medicine and surgical simulation. Altogether those developing areas will certainly revolutionize standard operating procedures in many surgical subspecialties, we are therefore confident that this Research Topic will become a valuable reference to our readers and that it will arouse their interest towards the future of surgery.
